# Potato Suberin Induces Differentiation and Secondary Metabolism in the Genus *Streptomyces*

**DOI:** 10.1264/jsme2.ME11282

**Published:** 2011-12-01

**Authors:** Sylvain Lerat, Martin Forest, Annie Lauzier, Gilles Grondin, Serge Lacelle, Carole Beaulieu

**Affiliations:** 1 Centre SÈVE, Département de Biologie, Université de Sherbrooke, Sherbrooke, Québec J1K 2R1, Canada; 2 Département de Chimie Université de Sherbrooke, Sherbrooke, Québec J1K 2R1, Canada

**Keywords:** cell wall, common scab, membrane, secondary metabolites, *Streptomyces scabiei*

## Abstract

Bacteria of the genus *Streptomyces* are soil microorganisms with a saprophytic life cycle. Previous studies have revealed that the phytopathogenic agent *S. scabiei* undergoes metabolic and morphological modifications in the presence of suberin, a complex plant polymer. This paper investigates morphological changes induced by the presence of potato suberin in five species of the genus *Streptomyces*, with emphasis on *S. scabiei. Streptomyces scabiei*, *S. acidiscabies*, *S. avermitilis*, *S. coelicolor* and *S. melanosporofaciens* were grown both in the presence and absence of suberin. In all species tested, the presence of the plant polymer induced the production of aerial hyphae and enhanced resistance to mechanical lysis. The presence of suberin in liquid minimal medium also induced the synthesis of typical secondary metabolites in *S. scabiei* and *S. acidiscabies* (thaxtomin A), *S. coelicolor* (actinorhodin) and *S. melanosporofaciens* (geldanamycin). In *S. scabiei*, the presence of suberin modified the fatty acid composition of the bacterial membrane, which translated into higher membrane fluidity. Moreover, suberin also induced thickening of the bacterial cell wall. The present data indicate that suberin hastens cellular differentiation and triggers the onset of secondary metabolism in the genus *Streptomyces*.

Suberin is among the most recalcitrant plant molecular structures in soils (31). Suberin forms a protective barrier in tissues such as woody stems, roots and underground storage organs which undergo secondary growth (10). This barrier controls the flux of water and also protects plant tissues against biotic diseases (29). Suberin is a biopolymer composed of polyaromatic and polyaliphatic domains linked by glycerol moieties (3). Microbial degradation of suberin is a process that is poorly characterized. Suberinases are poly-esterases that can depolymerize, at least partially, the lipidic polymer (16). Suberinases have been shown to be produced by some fungi belonging to the following genera: *Armillaria*, *Aspergillus*, *Coprinopsis* and *Fusarium*(16). There is also evidence that some actinomycetes produce suberin-degrading esterases. Esterase activity is induced in *Thermoactinomyces vulgaris*(10) and the plant pathogen *Streptomyces scabiei*(32) in the presence of suberin.

The genus *Streptomyces* belongs to the order of Actinomycetales, a division of Gram-positive bacteria that are characterized by a genome with a high G+C content. Their complex life cycle includes soil colonization by mycelial growth and terminates with sporulation. *Streptomyces* are known for producing a wide variety of biologically active secondary metabolites such as antibiotics; however, among the numerous species of the genus *Streptomyces*, a few have developed phytopathogenic traits, mainly relying on their ability to produce plant toxic secondary metabolites called thaxtomins (2). *Streptomyces scabiei* is the main causal agent of common scab, a severe disease which affects potato tubers and tap root crops (18).

The cellular response of *S. scabiei* exposed to suberin was investigated using a proteomic differential display technique. It was revealed that its presence up-regulated proteins related to the stress response, glycolysis, morphological differentiation and secondary metabolism (17). The effect of suberin on differentiation was corroborated by cultivating *S. scabiei* in the presence or absence of suberin (19). Suberin strongly stimulates aerial mycelium development in *S. scabiei* which, in the presence of cellobiose, can lead to the production of the secondary metabolites, thaxtomins (19).

Suberin is not the only biopolymer that influences differentiation and secondary metabolism in *Streptomyces*. For instance, chitin, the main polymer of insect cuticles and crustacean shells, modulates antibiotic biosynthesis and development in *Streptomyces coelicolor*(33). Since soil-dwelling streptomycetes can hydrolyse complex natural biopolymers, it was suggested that these polymers play a determinant role in the production of their bioactive molecules (33).

In the present paper, suberin is shown to affect development and secondary metabolite biosynthesis not only in *S. scabiei*, but also in the plant pathogen *S. acidiscabies*, as well as in saprophytic species such as *S. avermitilis*, *S. coelicolor* and *S. melanosporofaciens*. The molecular mechanisms responsible for the stimulation of differentiation are still unknown, but we demonstrate that suberin acts as a membrane and cell-wall perturbant in streptomycetes.

## Materials and Methods

### Suberin purification, bacterial cultivation and growth conditions

Suberin was obtained from potato peel (*Solanum tuberosum* ‘Russet’) and purified (15). Briefly, potato tubers were sliced and boiled for 20 min. The skin (suberin) was removed and flesh was roughly scraped away. The peel was then rinsed with tap water and residual flesh was digested overnight with cellulase (5 g L^−1^) and pectinase (1 g L^−1^) in 50 mM acetate buffer (pH 4.0). The peel was rinsed again with chloroform:methanol (2:1) and suberin purification was achieved using a Soxhlet extractor with chloroform as a solvent. Finally, suberin was dried and ground for 15 s in a coffee blender.

*Streptomyces scabiei* strain EF-35 (HER1481) was initially isolated in Quebec (Canada) from scabby potato tubers (9) and was used in all assays. Strains *S. acidiscabies* ATCC 49003, *S. avermitilis* ATCC 31267, *S. coelicolor* M145 (ATCC BAA-471) and *S. melanosporofaciens* EF-76 (ATCC BAA-668) were also used in the morphology and mechanical lysis experiments. Unless otherwise specified, bacteria were cultivated in liquid medium as follows. 10^7^ to 10^8^ spores were inoculated in 50 mL tryptic soy broth (TSB) and grown in a rotary shaker (250 rpm) at 30°C for 48 h. Bacteria were then centrifuged (10 min at 3,450×*g*) and resuspended in 5 volumes of sterile 0.85% NaCl. Volumes of 5 mL of this suspension were used to inoculate flasks containing 200 mL minimal medium (0.5 g L^−1^ asparagine, 0.5 g L^−1^ K_2_HPO_4_, 0.2 g L^−1^ MgSO_4_ and 5 mg L^−1^ FeSO_4_–7H_2_O) supplemented with 1% (w/v) soluble starch and 0% (control medium: CM) or 0.1% (w/v) suberin (suberin medium: SM). In order to collect suberin-free bacterial samples at the end of the experiment, suberin was placed in *ca*. 4×4 cm cotton pouches (200 mg suberin per pouch). Control flasks contained empty cotton pouches. Bacteria were grown at 30°C with shaking (250 rpm).

### Morphology of bacterial colonies on solid medium

Morphology of the five *Streptomyces* species tested in this study was determined as previously described (19). Briefly, 30–50 viable spores from each species were streaked on Petri dishes containing solidified (15 g L^−1^ agar) CM and SM. Petri dishes were incubated at 30°C for 5 d and representative colonies of each species and each treatment were photographed.

### Production of secondary metabolites

The production of characteristic secondary metabolites, thaxtomin A for *S. scabiei* and *S. acidiscabies*, actinorhodin for *S. coelicolor* and geldanamycin for *S. melanosporofaciens*, was assessed. These four strains were grown in CM and SM; for the growth of *S. scabiei* and *S. acidiscabies*, a starch/cellobiose combination (0.5% each) (19) was used instead of 1% starch since cellobiose is required for the production of thaxtomin A (12, 13). After 4 d, bacterial cultures were centrifuged (10 min, 3,450×*g*) and supernatants were decanted for quantification of metabolites. Pellets were dried (24 h at 50°C) and weighed to determine bacterial growth.

Thaxtomin A produced by *S. scabiei* and *S. acidiscabies* was purified as previously described (11) and quantified by HPLC Agilent 1260 Series (Agilent Technologies, Santa Clara, CA, USA) at 249 nm using a Zorbax SB-C18 column (Agilent Technologies). Abamectin (B_1a_) was extracted with ethyl acetate and quantified by HPLC at 246 nm (20). γ-Actinorhodin produced by *S. coelicolor* was quantified by spectrophotometry according to Kieser *et al.*(14). Geldanamycin produced by *S. melanosporofaciens* was purified by chloroform extraction and quantified by HPLC at 306 nm (4). This experiment was carried out in triplicate.

### Cell wall morphology of *Streptomyces scabiei*

The cell wall morphology of *S. scabiei* grown in the absence or presence of suberin was determined after 7 d of growth. Preparation of bacterial cells, the microscopy procedure and image analyses were carried out according to Miguélez *et al.*(25). Samples were examined with a Philips EM201 (FEI Company, Hillsboro, OR, USA) electron microscope at 60 kV and photographed on an Eastman Fine Grain Positive film 5302 (Eastman Kodak, Rochester, NY, USA). High-contrast photographic negatives were digitized with a HP Scanjet 6300C slide scanner and images were analyzed using the Image-Pro Plus v.4.5 software (Media Cybernetics, Elizabeth, IN, USA). Bacteria sliced in the middle of the cell, *i.e.*, with a well-defined cell wall and a clearly visible DNA zone in the centre, were selected for analysis. Pixel intensity from zones randomly selected in cell walls (40 zones per treatment) was measured. Cell wall thickness was also determined (25 walls per treatment).

### Bacterial resistance to mechanical lysis

The five *Streptomyces* strains were grown for 7 d in liquid CM and SM (using cotton pouches) containing 2% starch. Bacteria were collected by centrifugation, rinsed with 10 mM Tris-HCl (pH 8.3) and centrifuged again. Supernatants were thoroughly discarded and 300 mg bacteria (fresh weight) were resuspended in 1 mL Tris-HCl buffer. Resistance to mechanical stress was assessed using 0.5 mL of this suspension with 250 mg glass beads (100 μm diameter) using a bead beater (FastPrep FP-120; Thermo Fisher Scientific, Waltham, MA, USA) for 45 s (speed 4.5 m s^−1^). Samples were chilled on ice, centrifuged and supernatants were filtered. Lysis efficiency was evaluated by quantifying protein concentration of supernatants using Bio-Rad protein assay (Bio-Rad Laboratories, Hercules, CA, USA).

### Measurement of bacterial membrane fluidity of *Streptomyces scabiei*

Membrane fluidity of *S. scabiei* EF-35 bacteria collected from control and suberin treatments was determined. After 1 d of growth in CM and SM, bacteria were centrifuged, washed with 0.85% NaCl and resuspended in 0.85% NaCl to a concentration of 12.5 g L^−1^ bacteria. Membrane fluidity was assessed by an anisotropy test, based on the incorporation of 1,6-diphenyl-1,3,5-hexatriene (DPH) into bacterial membranes. The method described by Shinitzky and Barenholz (35) was used with minor modifications. Twenty microliters of 1 mM DPH (prepared in acetone and kept in the dark) (1) was added to 10 mL bacterial suspension to obtain a final DPH concentration of 2 μM (30). Suspensions were incubated in the dark for 2 h at room temperature with mild shaking. Bacteria were then washed with one volume of 0.85% NaCl, resuspended with exactly 10 mL of 0.85% NaCl and kept on ice. Anisotropy tests were performed using a spectrofluorimetry system equipped with PTI polarizers. Fluorescence of the DPH probe was measured with an excitation wavelength of 355 nm and an emission wavelength of 425 nm (22), from 10°C to 40°C by 5°C increments. Data were analyzed with Felix 32 v.1.1 software (Photon Technology International, London, ON, Canada).

In another experiment, membrane fluidity of *S. scabiei* EF-35 was determined over a 4-d period. Bacteria were grown in CM and SM (200 mL, four replicates per treatment) and 20 mL bacterial suspension was collected every day. Membrane fluidity was readily measured as described above at a temperature of 25°C.

### Determination of bacterial membrane fatty acid composition of *Streptomyces scabiei*

Membrane fatty acid composition of *S. scabiei* EF-35 bacteria grown in CM and SM was examined. Bacteria were pelleted by centrifugation, washed with 0.85% NaCl and lyophilized. Bacterial membrane fatty acids were extracted and methylated as in Moss (27), with modifications. Saponification was performed on 150 mg dried cells with 1 mL of 15% NaOH in 50% ethanol. Suspensions were incubated for 30 min at 100°C; samples were then cooled and brought to pH 2.0 with 6N HCl. Methylation of fatty acids was achieved after adding 3 mL of 14% BF_3_ to methanol and by incubating at 80–85°C for 20 min. After cooling, methylated fatty acids were extracted twice with one volume of petroleum ether/hexane (1:1) and evaporated to 1 mL with a flow of N_2_. Extracts were then washed with 0.3 N NaOH solution (26). The organic phase was transferred to a new tube and the solvent was completely evaporated by N_2_ flow. Residual H_2_O was eliminated by the addition of 80–100 mg Na_2_SO_4_. Tubes were stored under N_2_ at −20°C until analysis. Dried fatty acids were then dissolved in 100 μL hexane and separated using a gas chromatograph HP6890 (Hewlett-Packard, Mississauga, ON) equipped with a capillary column RTX-1 of 30 m × 250 μm × 0.25 μm (Restek, Bellefonte, PA, USA). Fatty acids were identified by comparing with the commercial standard mixes Bacterial Acid Methyl Esters Mix (Matreya, Pleasant Gap, PA, USA) and Supelco 37-component FAME Mix (Sigma, St-Louis, MO, USA).

## Results

### Suberin promotes secondary growth and production of secondary metabolites in *streptomycetes*

**Fig. 1:**
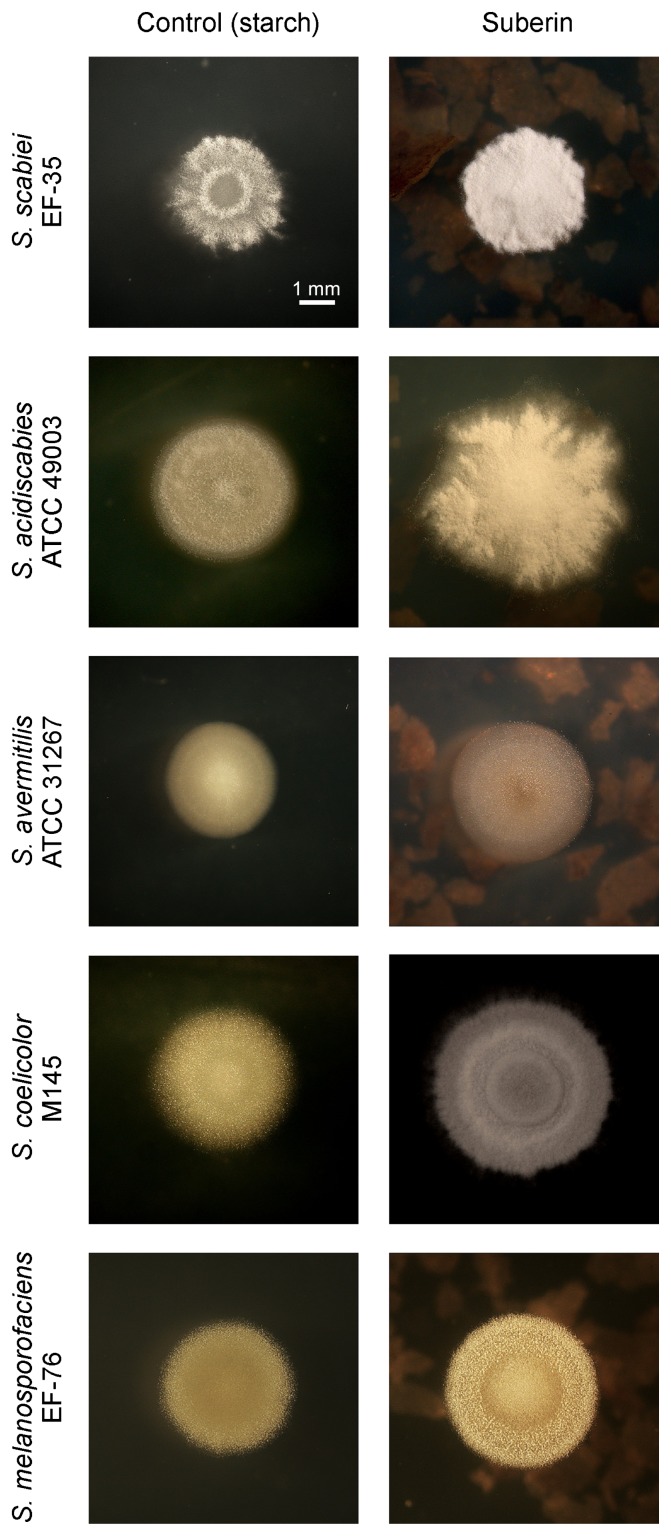
Typical morphology of isolated colonies of *Streptomyces scabiei* EF-35, *S. acidiscabies* ATTC 49003, *S. avermitilis* ATTC 31267, *S. coelicolor* M145 and *S. melanosporofaciens* EF-76 after 5 d of growth on solid minimal starch (1%) medium, complemented or not with 0.1% suberin.

The composition of growth media noticeably influenced the morphology of the five *Streptomyces* strains tested in this experiment (Fig. 1). As previously described (19), moderately hairy colonies were observed when *S. scabiei* EF-35 was grown on starch medium while suberin triggered the onset of secondary metabolism (formation of hairy colonies). Suberin also strongly stimulated the production of aerial hyphae in *S. acidiscabies* ATCC 49003, *S. coelicolor* M145 and *S. melanosporofaciens* EF-76 when compared to minimal starch medium (CM). In *S. avermitilis* ATCC 31267, suberin only moderately stimulated aerial growth, while suberin-deprived colonies were bald (Fig. 1).

In liquid minimal medium, the presence of suberin significantly stimulated the growth of all *Streptomyces* spp. tested (Table 1). Furthermore, the production of secondary metabolites typically synthesized by these strains was strongly promoted by the presence of the plant polymer in *S. scabiei*, *S. acidiscabies*, *S. coelicolor* and *S. melanosporofaciens*. In the absence of suberin, neither thaxtomin A nor geldanamycin was detected in media inoculated with *S. scabiei* and *S. melanosporofaciens*, respectively, while metabolite production was significantly limited in flasks inoculated with *S. acidiscabies* and *S. coelicolor* (Table 1). Substantial production of unidentified secondary metabolites was also detected from HPLC chromatograms of *S. acidiscabies* and *S. melanosporofaciens* grown in the presence of suberin (Fig. S1). Abamectin production by *S. avermitilis* could not be detected in the presence or absence of suberin; however, three-dimensional HPLC profiles showed a strong increase in the production of various unidentified molecules in the presence of suberin (Fig. S1).

### Suberin alters cell wall morphology

**Fig. 2:**
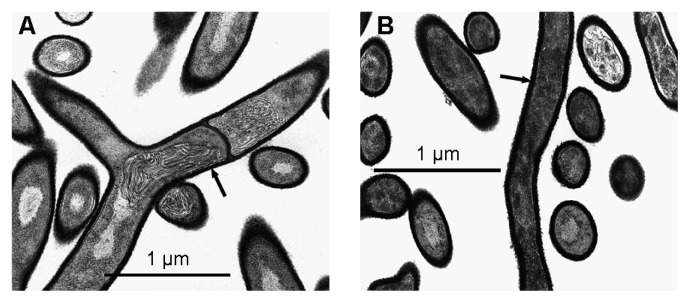
Electron microscopy images of *Streptomyces scabiei* EF-35 after 7 d of growth in minimal medium (A) or in suberin-supplemented medium (B), at a 35,590× magnification. Arrows show thicker cell wall in bacteria grown in the presence of suberin.

The presence of suberin in growth medium induced morphological changes in *S. scabiei* EF-35. These modifications were clearly visible by electron microscopy after 7 d of growth. The cell walls of bacteria that had been exposed to suberin contained a high quantity of electron-dense material (Fig. 2). Cell-wall density of bacteria grown in the presence of suberin (90.5±6.7 pixels) thus appeared significantly higher than cell-wall density of control bacteria (75.2±6.9 pixels; *P*<0.0001, *t*-test). Image analyses also revealed that cell walls were thicker in suberin-treated bacteria (46.6±8.8 nm) than in control bacteria (36.6±6.7 nm; *P*<0.0001, *t*-test).

### Bacteria grown in the presence of suberin showed higher resistance to mechanical lysis

Protein content of supernatants obtained after mechanical lysis of bacterial suspensions was significantly higher for bacteria grown for 7 d in CM than in SM, in all *Streptomyces* strains tested (Table 2).

**Fig. 3:**
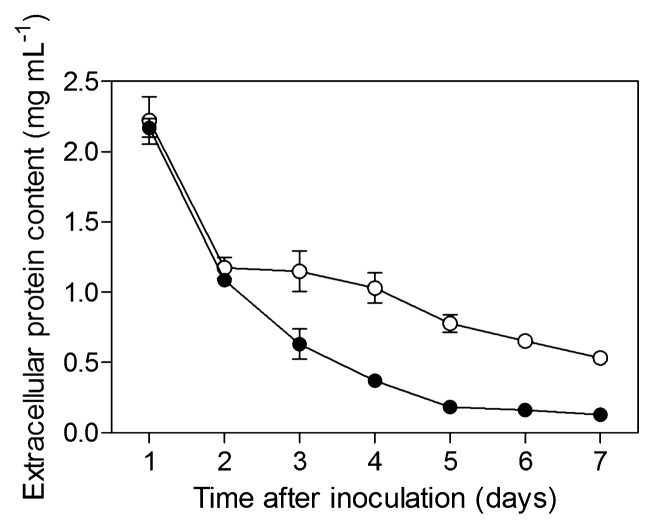
Extracellular protein contents (±SD) released by mechanical lysis of *Streptomyces scabiei* EF-35 grown in minimal control medium (open circles) and in suberin-minimal medium (solid circles) over a 7-d period.

An additional experiment was consequently conducted to assess resistance to mechanical lysis over a 7-d period. *S. scabiei* was grown in CM and SM and resistance to mechanical lysis was measured every day, as described above. The amount of protein released by mechanical treatment was similar for both experimental conditions after incubation periods of 1 and 2 d (Fig. 3); however, from day 3 to day 7, resistance to mechanical lysis was significantly higher (*i.e.*, protein concentration was lower) in bacteria grown in the presence of suberin (Fig. 3).

### Membrane fluidity and fatty acid composition

**Fig. 4:**
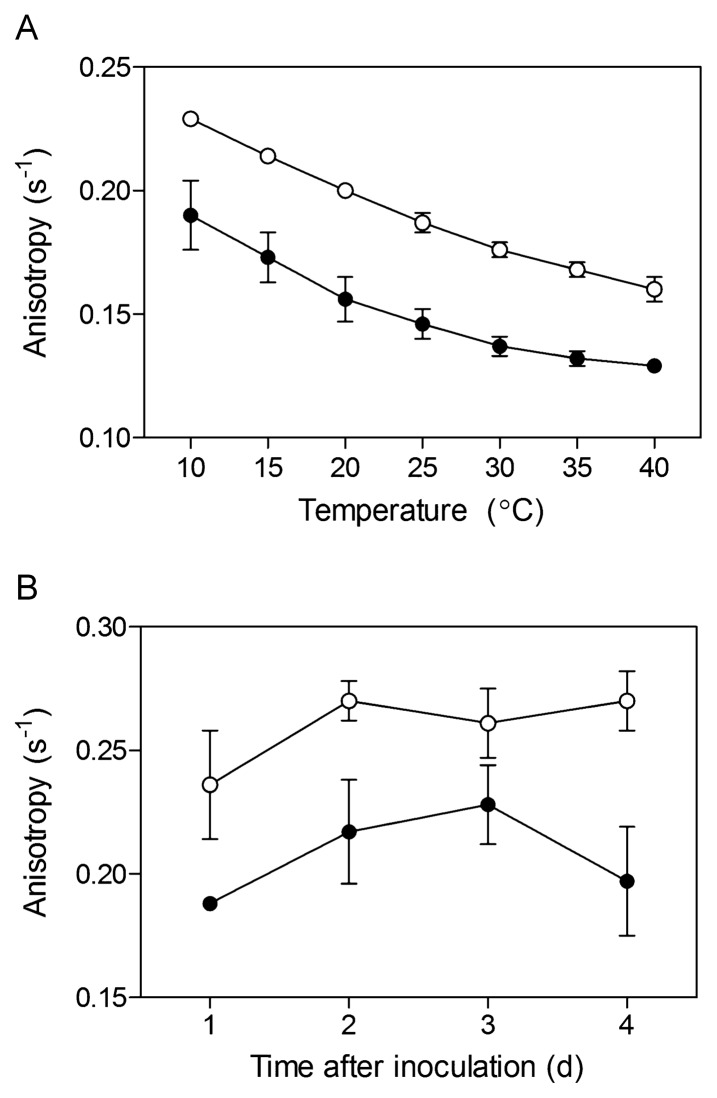
Anisotropy (±SD) of the DPH probe incorporated into the membrane of *Streptomyces scabiei* EF-35 grown in minimal medium (open circles) and in suberin-supplemented medium (solid circles) after 1 d of incubation, as a function of temperature (A); and measured at 25°C over a 4-d period (B).

Anisotropy measurements performed with the DPH probe, 1 d after inoculation in minimal medium, revealed that membrane fluidity of *S. scabiei* was significantly higher (*i.e.*, anisotropy was lower) in bacteria grown in the presence of suberin than in control bacteria. This pattern was observed at all temperatures tested (Fig. 4A). The greater membrane fluidity of bacteria grown in the presence of suberin was maintained over the 4 d time course performed at 25°C (Fig. 4B).

The analysis of fatty acid composition revealed that the membranes of *S. scabiei* contained a majority of two branched-chain (*iso*-16:0 and *anteiso*-15:0), an unsaturated (16:1 [9] *cis*, *i.e.*, palmitoleic acid) and a straight-chain (16:0, *i.e.*, palmitic acid) fatty acids (Table 3). Differences between bacteria grown in the absence or presence of suberin were observed after 1 d of growth in minimal medium. Suberin induced a higher proportion of total branched-chain fatty acids. The abundance of two of these, *iso*-16:0 and *anteiso*-17:0, increased significantly in the presence of suberin while the abundance of *iso*-15:0 and *anteiso*-15:0 remained unchanged; however, suberin induced a significantly lower proportion of unsaturated acids. No variation in the proportions of straight-chain fatty acids was observed (Table 3).

## Discussion

Suberin is a polymer recalcitrant to microbial degradation in nature. Unambiguous evidence for the presence of suberin in soil organic matter has been revealed by different groups (24, 28, 31). Only rare studies have investigated the biochemical mechanisms associated with suberin degradation (16). Nevertheless, some authors have suggested that actinomycetes might be involved in the degradation process (10, 32). All *Streptomyces* strains used in this study showed better growth in the presence of the polymer. This enhanced growth is probably not the effect of the utilization of suberin constituents as a carbon source (suberin concentration was low), but would rather result from an increase in membrane fluidity that facilitates the transport of nutrients and waste products (5).

The fatty acid monomers associated with the suberin structure act as membrane perturbants of phospholipid vesicles (8). Here, suberin affected both the membrane composition and fluidity of living *S. scabiei* cells. The anisotropy test unequivocally showed that in the *S. scabiei* EF-35 membrane, fluidity was overall higher in bacteria grown in the presence of suberin. *Iso* and *anteiso* branched-chain fatty acids in the bacterial membrane generally contribute to its fluidity (6, 34) and the proportions of branched-chain fatty acids increased in bacterial cells grown in the presence of the polymer. On the other hand, unsaturated fatty acids also have the ability to increase membrane fluidity (6, 7), but their proportions decreased in suberin-treated membranes, showing that adjustment of membrane fluidity is a complex mechanism. This decrease in the proportions of unsaturated fatty acids may, however, represent a protection mechanism against phenolic compounds present in the suberin polymer. Denich *et al.*(6) stated that saturated fatty acids help in preventing the access of phenol molecules to the membrane interior.

The presence of suberin in the growth media of the five *Streptomyces* species tested conferred relative protection against mechanical stress, suggesting that this plant polymer triggers changes not only in the bacterial membrane, but also in the cell wall. This was demonstrated in *S. scabiei* since cell walls were thicker in bacteria grown in the presence of suberin. The cell wall also appeared to contain more electron-dense material. The increase in cell-wall thickness may explain the higher resistance of *S. scabiei* to mechanical lysis. Once again, the presence of phenols in suberin may be responsible for the higher thickness of cell walls associated with bacteria grown in the presence of suberin. It was suggested that a high number of peptidoglycan layers may effectively be caused by the exposure of bacterial cells to environmental stresses such as phenols (21).

In the present study, potato suberin altered the development of five bacterial species of the genus *Streptomyces* (*S. scabiei*, *S. acidiscabies*, *S. avermitilis*, *S. coelicolor* and *S. melanosporofaciens*). The general morphological patterns of isolated colonies of the five *Streptomyces* species used here point toward the capacity of suberin to promote cellular differentiation. On solid minimal medium, colonies of the five species tested here presented a hairy morphotype in the presence of suberin (although this phenotype was only slightly visible in *S. avermitilis*), while in the absence of the biopolymer, colonies were bald, or to some extent hairy. The onset of morphological differentiation attributable to suberin concurs with the production of secondary metabolites whose presence, after 4 d of growth in liquid minimal medium, boosted the synthesis of characteristic phytotoxins and antibiotics by *S. scabiei*, *S. acidiscabies*, *S. coelicolor* and *S. melanosporofaciens*. Although no abamectin was produced by *S. avermitilis* under the tested conditions, the production of other unidentified metabolites was apparently stimulated by the presence of suberin. Abamectin is only one secondary metabolite potentially produced by *S. avermitilis* and other antibiotics may also be synthesized by this bacterium (20).

It has been speculated that complex polymers could influence the development and production of bioactive molecules by soil-dwelling streptomycetes (33). For instance, *N*-acetylglucosamine and cellobiose, the main degradation products of chitin and cellulose, lock *S. coelicolor*(33) and *S. scabiei*(19) in the vegetative state. The effect of cellulose and cellooligosaccharides on morphological development has also been described in *S. griseus*(23). Interestingly, it has been demonstrated that suberin counteracts the effect of cellobiose and could promote morphological differentiation, even in the presence of the disaccharide (19). In *S. coelicolor*, environmental stresses trigger cell morphological differentiation associated with secondary metabolism (36) and it was shown that suberin is perceived by streptomycetes as a stress factor (17).

The data presented in this paper not only confirm previous observations suggesting that suberin triggers the onset of secondary metabolism (17, 19) but also bring to light new characteristics of the changes induced by the presence of suberin. When exposed to this plant polymer, streptomycetes undergo profound morphological modifications. While mechanisms linked to suberin biodegradation in streptomycetes are still largely unknown, determining which suberin constituents trigger differentiation is challenging. Characterization of the secretome of *Streptomyces* species grown in the presence of suberin is in progress.

## Supplementary Material



## Figures and Tables

**Table 1:** Bacterial growth and production of typical secondary metabolites by five *Streptomyces* species grown for 4 d in MM in the absence or presence of suberin

	Dry mycelial weight (mg±SD)		Metabolite production (μg mg DW^−1^±SD)^a^	
		
	control	+ suberin^b^	control	+ suberin^b^
*S. scabiei*	28±4	90±3***	n.d.	3.61±0.14***
*S. acidiscabies*	60±3	88±2***	0.05±0.00	1.44±0.42**
*S. avermitilis*	27±4	134±3***	n.d.	n.d.
*S. coelicolor*	38±1	64±4***	0.35±0.20	1.22±0.45*
*S. melanosporofaciens*	41±4	69±6**	n.d.	1.32±0.79***

Values are the means of three replicates.

a Metabolites assayed were thaxtomin A for *S. scabiei* and *S. acidiscabies*, abamectin for *S. avermitilis*, γ-Actinorhodin for *S. coelicolor* and geldanamycin for *S. melanosporofaciens*.

b Values from suberin medium are significantly different from control at *: *P*<0.05, **: *P*<0.01 and ***: *P*<0.001 (*t*-test).

n.d.: not detected; detection limits were 0.05 μg, 0.05 μg and 0.1 μg of total thaxtomin A, abamectin and geldanamycin, respectively.

**Table 2:** Proteins (mg mL^−1^±SD) released by mechanical lysis performed on five *Streptomyces* species grown for 7 d in the absence or presence of suberin

	*S. scabiei*	*S. acidiscabies*	*S. avermitilis*	*S. coelicolor*	*S. melanosporofaciens*
Control	0.26±0.05	0.75±0.06	0.71±0.06	1.05±0.10	2.23±0.11
Suberin	0.15±0.05	0.48±0.07	0.29±0.04	0.77±0.08	1.52±0.09
*P* value (*t*-test)	0.0005	0.0013	0.0001	0.0223	<0.0001

Values are the means of four replicates.

**Table 3:** Membrane fatty acid composition of *Streptomyces scabiei* EF-35 grown for 1 d in minimal medium in the absence or presence of suberin

Fatty acids	Control (%±SD)	Suberin (%±SD)
Branched-chain**	51.0±3.4	55.2±0.6
*anteiso*-13:0	1.7±0.3	1.7±0.3
*iso*-13:0	2.1±0.1	2.1±0.1
*iso*-14:0	5.4±0.4	5.0±0.3
*iso*-15:0	4.2±0.4	4.22±0.23
*iso*-16:0**	17.5±1.9	20.0±0.2
*anteiso*-15:0	11.3±0.3	12.2±0.5
*iso*-17:0	2.9±0.2	2.7±0.1
*anteiso*-17:0**	6.0±0.4	7.2±0.0
Unsaturated*	18.8±2.5	15.5±0.3
16:1 (9)cis*	16.4±3.1	13.0±0.1
*iso*-17:0	2.4±0.6	2.5±0.2
Straight-chain	30.2±0.9	29.3±1.4
14:0	1.2±0.3	1.1±0.3
14:0 3-OH	5.6±0.4	5.3±0.1
15:0	3.3±0.6	3.2±0.7
16:0	15.3±1.7	14.3±0.5
17:0 cyclopropane (9, 10)	3.7±1.1	4.2±0.4
18:0	1.1±0.1	1.2±0.0

Values are the means of six replicates (three replicates of two repeats). ANOVA; *: *P*<0.05, **: *P*<0.01.
