# Supplementary material for: Potato Suberin Induces Differentiation and Secondary Metabolism in the Genus Streptomyces
Source: Microbes Environ. 2011 Dec 1;27(1):36–42. doi: 10.1264/jsme2.ME11282 (PMC4036036; doi:10.1264/jsme2.ME11282)

Fig. S1. Three-dimension [*x*: time (min), *y*: wavelength (nm), *z*: peak height (mAu)] HPLC chromatograms of secondary metabolites produced by *Streptomyces scabiei* EF-35 (A), *S. acidiscabies* ATTC 49003 (B), *S. avermitilis* ATTC 31267 (C) and *S. melanosporofaciens* EF-76 (D) after 4 d of growth in control and suberin media.

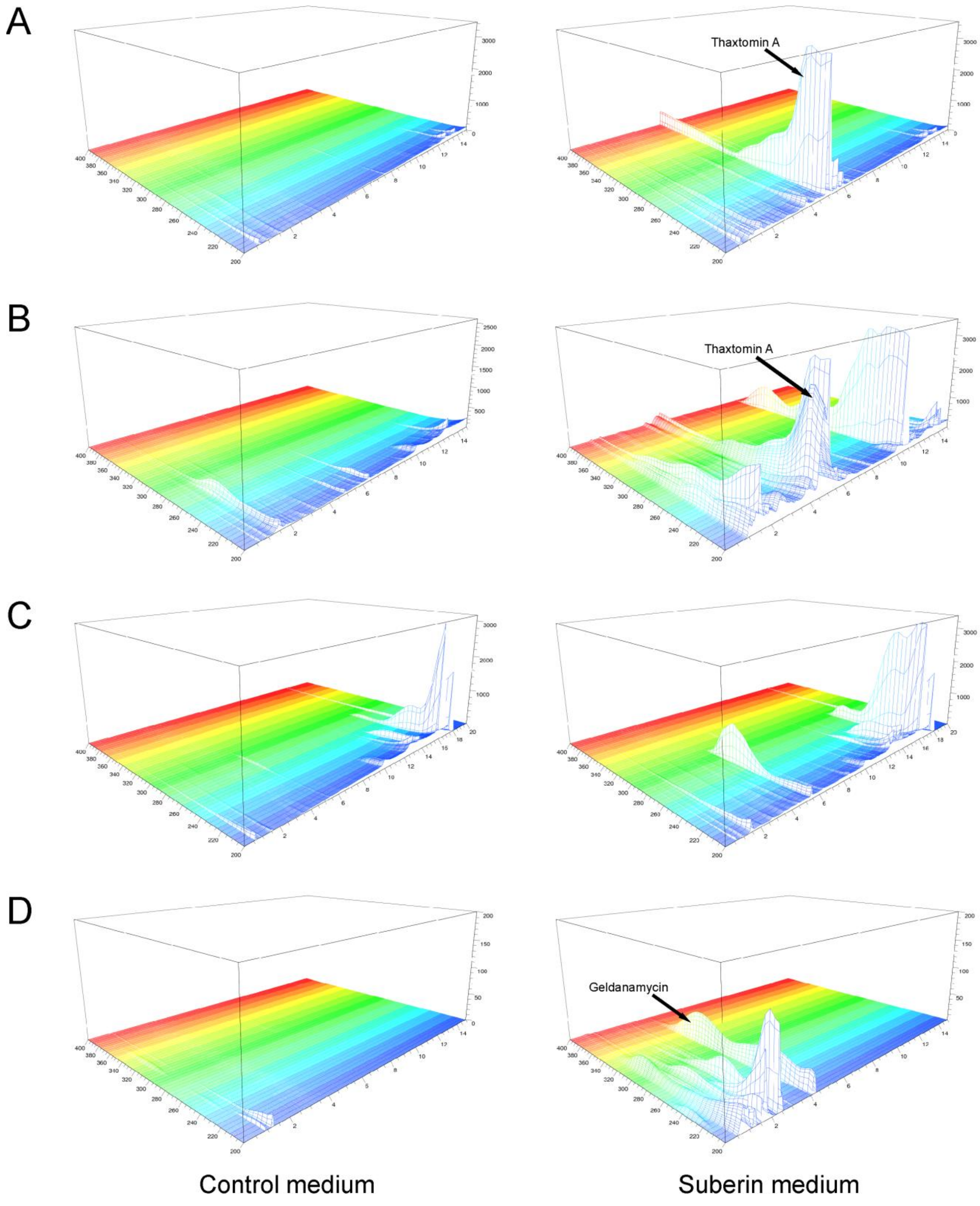

Supplement: Supplementary file 1 [file 27_36_s1.pdf]
